# Beyond yield: Unveiling farmer perceptions and needs regarding weed management in Bangladesh

**DOI:** 10.3389/fbioe.2024.1410128

**Published:** 2024-10-11

**Authors:** Md Mirajul Islam, Md Mizanur Rahman, Shashanka Shekhar Sarker, Md Nazrul Islam, Fahmid H. Bhuiyan, Mst Salma Khanam, Iftekhar Alam

**Affiliations:** ^1^ Plant Biotechnology Division, National Institute of Biotechnology, Dhaka, Bangladesh; ^2^ Department of Agronomy, Patuakhali Science and Technology University, Dumki, Patuakhali, Bangladesh

**Keywords:** baseline survey, herbicide, rice, toxicity, weed management representative site, number of surveyed villages and shops

## Abstract

More than 3.5 billion people depend on rice for more than 20% of their daily calories. Globally, Bangladesh is the third largest rice producer. With 171 million people, Bangladesh is also among the top consumers. Local rice production not only affects the country’s food security but also influences the global rice trade. A large yield gap has been reported due to weeds. Traditional hand weeding is very costly because of labor shortages resulting from industrialization. Limited data showed a higher yield and profits when using herbicides. However, quantitative data on various aspects of weed management and associated issues representing the country’s variable rice ecosystem, which is characterized by 30 agroecological zones, are lacking. We collected data on weed management practices from 865 farmers and 69 agrochemical shops covering all 30 agro-ecological zones (AEZs) through a structured survey. We observed a significant regional variation among various parameters. Approximately 82% of farmers use herbicides, and few rely solely on either manual weeding or herbicides. Pre-emergence herbicides are the predominant. Application procedures are almost the same across the country. Although 40% of farmers had secondary and higher-level education, most depend upon local sellers’ suggestions rather than reading the product label regarding the dose. Few farmers consider herbicides hazardous, and respondents rarely perceive any environmental impact. Pyrazosulfuron ethyl (35%) and acetochlor-containing bensulfuron methyl (27%) are the most-used chemical species. Approximately 45% of farmers observed that herbicides suppress early seedling growth. Additional fertilizer is required to compensate for this. Multiple weed species that are difficult to control through presently used herbicides were noted in all AEZs. Around 64% of farmers observed that herbicide application contributes to higher yields as a function of timely weeding. Cost comparisons showed that high labor prices will make rice cropping unprofitable in most parts of the country if herbicides are eliminated. Clear adverse effects of pre-emergence herbicides on early crop growth implied the potential benefits of broad-spectrum herbicide-tolerant genetically engineered (GE) rice to sustain the country’s food security. Additionally, such GE rice could incentivize the adoption of alternate wet and dry irrigation methods, leading to water and cost savings.

## 1 Introduction

In well-managed agroecosystems, weeds grow spontaneously and challenge cultivated plants in various ways, eventually reducing crop yield. In general, weeds have evolved with a much higher ability to survive and flourish than domesticated crops. Competition for resources such as nutrients, light, and spaces from a shared, finite pool by neighboring individuals is the leading cause of weed-induced yield loss. Recent evidence also suggested that crop plants negatively respond to weeds (and reduce yield) even when resources are not limited (Linu and Girija, 2020; Gu et al., 2022; Horvath et al., 2023). Thus, effectively controlling weeds is essential for sustainable crop cultivation.

Rice is the most significant and widely cultivated cereal crop in tropical and subtropical regions worldwide. It occupies approximately 10% of all arable land, providing a staple for more than 3.5 billion people. The global population is projected to reach 9 billion by the year 2050, leading to concerns about food security. Rice-dependent countries seek their future food security through a projected increase in rice productivity—specifically, by yield increment per unit area of land (Arifin et al., 2021; Kabir et al., 2015; ICAR, 2013).

Most cultivated rice is grown in tropical and subtropical regions in the warm season and thrives in waterlogged soil. Such an environment is highly favorable for many persistent weeds. Shallow flooded land maintained during early seedling growth also favors the development of numerous weeds. Thus, weeds are among the leading causes of reduced rice productivity and impose a significant management cost. In addition, climate change threatens weed management in cropping systems worldwide (Ramesh et al., 2017; Marambe and Wijesundara, 2021). Advantaged from their more efficient physiological traits, climate change often favors prolonged growing seasons. Reduced water availability due to recurrent and unforeseen droughts would alter the competitive balance between crops and some weed species, intensifying the crop-weed competition pressure.

In rice cultivation, competition from weeds is one of the main biophysical yield constraints (Waddington et al., 2010). A study showed that approximately 12%–18% yield loss occurred because of weed infestation in upland and lowland rice fields (BRKB, 2011). Severe yield loss has been reported in various climates, up to 49% in the Sahel (Johnson et al., 2004) and 50% in Indonesia, irrespective of rice production system or season (Zoschke, 1990). Other studies estimated that uncontrolled weeds caused rice crop losses of 40% in China before widespread herbicide adoption. Severe yield loss has also been reported in other rice-growing countries, including India (national average ∼25.6%; Hossain et al., 2020).

Research on weeds and weed management in Bangladesh is less extensive than in other countries. On average, the gap in rice yields in farmers’ fields due to poor weed control was estimated to be 43%–51% (Rashid et al., 2012), and the yield gap was as high as 1 t/ha, with 30% of farmers losing more than 500 kg/ha (Ahmed et al., 2001). However, it is possible to reduce the cost of rice production and the yield gap by improving weed management technologies.

Bangladesh is the third largest rice producer after China and India (FAOSTAT, 2023). Most recent statistics show that the consumption of rice and products is 260 kg/capita/year, the highest in Asia and much higher than the global average (81 kg/capita/year; FAOSTAT, 2023). Bangladesh is in a deficit of rice with a significant import value. Food security in Bangladesh is synonymous with rice productivity in a given year. Due to the large population and extremely high dependency on the rice-centric diet, local rice shortages influence the global rice trade. To satisfy its 171 million population (and annual growth of two million), Bangladesh must increase rice yield from the current 2.74 t/ha to 3.74 t/ha over the next 20 years (BRKB, 2011).

Traditionally, hand weeding is the most widely used practice in Bangladesh and many developing countries. Industrialization has drawn a large labor force away from agriculture, leading to a noticeable shortage of workers in the agricultural sector. The introduction of herbicides offers reduced labor requirements for weed control. One study showed that herbicide reduces weed control time to 84 person-hours/ha compared to 590 person-hours/ha, considering the requirement of two rounds of hand weeding (Mazid et al., 2006). Herbicides are a newer introduction for weed control and appear as a potential solution for reducing yield losses caused by weeds and meeting the growing population’s demand for food (Kashem et al., 2009). However, proper species, dosage, and timely application are essential for economic benefits and food security.

Herbicides are high-tech solutions that can negatively impact crop growth if not applied properly. The choice of weed management practices depends on the weed composition, availability of tools, and workforce. Farmers adopt weed control methods based on availability and cost-effectiveness. Thus, understanding the extent of different weed management practices across all agroecological zones is essential for effective policy making.

Bangladesh is divided into 30 diverse agroecological zones. Soil type, topography, and water availability are different. Hence, agricultural practices, cropping patterns, and the occurrence of weeds are also diverse. Sporadic studies showed the potential benefits of herbicides (Hossain, 2015; Mia et al., 2021). However, comprehensive nationwide data on weed control practices, herbicide usage, advantages and disadvantages, and potential economic benefits are currently unavailable. The disadvantages of weed control and associated costs were not reported. A countrywide survey of present weed management practices would enable the scope of future interventions and analysis of economic aspects of weed control, providing a basis for the strategic use of manual weeding or herbicides in managing diversified weeds across various agroecological systems (Beltran et al., 2011; Rodenburg et al., 2019; Boyd and Reuss, 2022).

This study aimed to establish baseline data on identifying the current diversified practices in weed management in Bangladesh. It will measure the relative contribution of different methods, their cost-benefits, contribution to productivity enhancement, and accessibility of farmers to herbicides. It will also consider perceived drawbacks, farmers’ knowledge, and accessibility. Additionally, a list of weeds showing resistance to herbicides identified by farmers under current rice farming practices will be included. We aim to assess the current knowledge level, attitudes, and practices of Bangladeshi farmers regarding the safe use of herbicides. Data-based decisions would contribute to an improved weed management strategy, regional needs, and the possibility of herbicide-tolerant GMOs. Field information is crucial to understanding the likelihood of placing GMOs in existing weed control strategies.

## 2 Materials and methods

### 2.1 Area and farmer selection

Bangladesh comprises 30 agroecological zones (AEZ) with distinct ecology and cropping patterns. This farmer survey was conducted among randomly selected rice farmers from all 30 AEZs. Location information of survey sites was taken using a portable GPS reader with the assistance of Google Maps. Stratified random sampling was chosen to ensure that the sample reflects the diversity of AEZs and agricultural practices across the country. In each AEZ, we interviewed farmers in multiple village communities to provide further randomization. Commonly used standard procedures (Krejcie and Morgan, 1970; Daniel, 1999) were considered regarding sample size. A minimum of 20 farmers were selected from each of the 30 agroecological zones for the survey, except for AEZ-24, where data on 10 farmers were obtained due to the smaller population size. The zones are characterized based on definite attributes such as soil physiography (soil parent materials and landforms of a particular area), hydrology (water holding capacity of soil and the water level of agricultural land), season, soil types, and tidal activity. The survey also included data on cropping patterns and the sources of irrigation for rice cultivation. Characteristics and data of the 30 AEZ of Bangladesh are given in Table 1.

**TABLE 1:** Basic survey information: agroecological zones (AEZ), GPS coordinate of the representative site, number of surveyed villages and shops.

AEZ	GPS coordinate	No. of nearby villages	No. of shops	Zone description
1	25.823588, 88.393359	3	2	Old Himalayan piedmont plain
2	26.103672, 89.127459	3	2	Active Tista floodplain
3	25.091179, 88.873756	2	4	Tista meander floodplain
4	24.4161120, 895445217	2	2	Karatoya-Bangali floodplain
5	24.5315981, 89.0417007	2	2	Lower Atria basin
6	24.789749, 88.705645	2	2	Lower Purnabhaba floodplain
7	24.890149, 89.571578	2	3	Active Brahmaputra-Jumana floodplain
8	24.315065, 90.164876	1	2	Young Brahmaputra floodplain and Jamuna floodplain
9	24.760292, 90.248920	2	2	Old Brahmaputra floodplain
10	23.758134, 88.936266	2	2	Active Ganges floodplain
11	23.0688162, 89.0791822	2	3	High Ganga river floodplain
12	23.571218, 89.800433	1	3	Low-high Ganges river floodplain
13	22.7683139, 89.5953063	2	2	Low Ganges river floodplain
14	22.969923, 89.815824	2	3	Gopalgonj-Khulna bil
15	23.499138, 90.424097	2	2	Arial bil
16	23.702475, 90.719734	3	2	Middle Meghna river floodplain
17	23.513068, 89.135748	1	3	Lower Meghna river floodplain
18	22.454336, 90.819306	2	3	Young Meghna estuarian floodplain
19	22.9957533, 90.1105094	3	3	Old Meghna estuarian floodplain
20	24.915433, 91.824180	1	2	Eastern Surma Kushyara floodplain
21	25.102277, 91.195001	1	2	Sylhet basin
22	24.415787, 91.428516	2	2	Northern and eastern piedmont plains
23	21.420553, 92.058327	3	2	Chittagong coastal plain
24	20.622227, 92.325893	1	0	Martin’s coral island
25	24.9661833, 89.2816454	2	3	Level Barind tract
26	24.566243, 88.365809	2	2	High Barind tract
27	25.808760, 89.037603	3	2	Northeastern Barind tract
28	24.028235, 90.362919	2	2	Madhupur tract
29	23.168823, 92.203608	3	3	Northern and eastern hills
30	23.959548, 91.176951	1	2	Akhaura terrace

### 2.2 Survey design

Farmers growing rice on at least 0.33 acres of land were included in this survey. An effort was made to choose farmers representing the average land size. In most cases, we interviewed at least 20 farmers from each agroecological zone. The survey was carried out during 2021–2023 by using a structured questionnaire. Primary information collected from each farmer in each site includes (1) zone and area name, (2) gender of participating farmers, (3) participant age, (4) educational qualification, (5) pre-cultivation rice-growing environment of the participating farmer, (6) cultivated rice varieties, (7) land size of respondents, and (8) number of crops per year (cropping pattern). Follow-up questions were: (1) what kind of weed management strategies did they apply (hand weeding/mechanical weeding/herbicide application/or combination, (2) what number of weed management intervention sessions are needed during a season, (3) in which stage of weed growth is herbicide applied (pre-emergence/post-emergence), (4) what kind of herbicide is used, its availability and the price of herbicide in local market, (5) years of herbicide adoption, (6) herbicide application procedure (spray/mixing with fertilizers/others), (7) source of information on type and dose (product label/extension workers/neighbor/colleague/shopkeeper/other), and (8) knowledge of active ingredients of herbicide. We also followed up on some questions for farmers who did not adopt herbicides. These were (1) reasons behind not using herbicides and (2) which issue needed to be addressed for the adoption of herbicides. In this survey, hand weeding refers to the practice of manually uprooting weeds or using any combination strategy that farmers employ to remove weeds by hand.

### 2.3 Market survey

An additional survey was conducted at marketplaces regarding the accessibility of herbicides to farmers. We included retail agrochemical shops from all AEZs. At least two agrochemical shops were surveyed in each zone, and available herbicides were listed. Other collected information includes brand name, active ingredients, price, seasonal variation on availability, company information, and dose recommendations on the label.

### 2.4 Yield observation and cost analysis

To assess the effects of herbicide adoption on rice productivity, we asked farmers how much yield increment they observed. The analysis was conducted based on their perception of increasing rice yield. Cost analysis was performed on different aspects of weeding and yield. We collected data from each farmer on weed control costs in the entire cultivation period, including labor wage, herbicide price, and application cost. All prices were converted from local currency to US dollars ($).

### 2.5 Farmers’ response to herbicide impact and future intervention

We collected data from farmers’ observations regarding the impact of herbicides. The questions include (1) any impact on crop yield by using herbicide application, (2) any adverse effect on rice or soil, (3) any health issues they noticed while using herbicides, (4) whether a herbicide is less harmful than a pesticide, (5) any side effects they observed during herbicide application, (6) is there any weed species that cannot be effectively controlled by regular herbicides, (7) is the alternate wet and dry (AWD) method of irrigation useful, and (8) knowledge of GMO and whether herbicide-tolerant rice is helpful for them.

### 2.6 Statistical analysis

Descriptive analysis was performed for weed management practices, weeding timing and frequency, herbicide types, herbicide prices, and information sources of herbicide use. Point-biserial correlation was used to find the relationship between herbicide adoption and a farmer’s total land size. Pearson chi-square (X^2^) test of independence variable was performed to determine whether there were any significant relationships among the data of herbicide use and some other factors (hand weeding, cultivation experience, education, yield, the negative impact on rice, human health, and environment). A one-way ANOVA test was performed to find the cost difference regarding weed management in our studied AEZs. A paired sample *t*-test was conducted to determine the effect of herbicide adoption on reducing costs. The latent structure of the weed management strategy (WMS) was examined using principal component analysis with varimax rotation. Multinomial logistic regression was performed to assess the impact of several factors affecting respondents’ adoption of herbicides. The model contained seven independent variables (total cost of herbicide, total cost of weed management, number of herbicide applications, number of hand weeding sessions, whether there is any negative effect of herbicide on rice, do herbicides increase yield, and any negative effect of herbicide on the environment). All the data were analyzed using IBM SPSS Statistics software, version 25.

## 3 Results

### 3.1 Location and farm characteristics

Bangladesh comprises 30 distinct AEZs, all of which were considered in this survey. Climatic parameters and natural resource characteristics are diverse and influence various aspects of agriculture. AEZs are distinct in seasonality, topography, soil type, soil fertility, drainage, temperature, availability of both surface and groundwater, and flood patterns. Cropping patterns are, therefore, different. Being positioned around the Tropic of Cancer, sunshine hours and intensity did not vary much except for microclimates such as open coastlines and hilly areas. The altitude of the rice production areas ranged from sea level to 268 feet above sea level. However, 57% of our survey sites ranged from 3 to 60 feet. A summary of the characteristics is provided in Table 2.

**TABLE 2:** Cropping pattern of the surveyed area in 30 agroecological zones (AEZs). Multiple cropping patterns were observed in some AEZs. The numbers of respondents are given for water availability. Timeline may vary by 2–4 weeks, depending on local situations.

Zone	Elevation (feet)	Pattern	Jan	Feb	Mar	Apr	May	Jun	Jul	Aug	Sep	Oct	Nov	Dec
AEZ-1	210–220	1	Fallow		Irrigated rice (20)				Fallow					
		2	Vegetable (20)		Irrigated rice (20)									Vegetable (20)
AEZ-2	223–233		Tobacco/Maize (14)			Irrigated rice (20)				Rain-fed rice (20)				Tobacco /maize (14)
AEZ-3	130–140			Irrigated rice (20)					Rain-fed rice (20)					
AEZ-4	27–33			Irrigated rice (20)						Rain-fed rice (20)				
AEZ-5	70–80		Fallow	Irrigated rice (24)				Fallow						
AEZ-6	79–85		Vegetable (18)		Irrigated rice (40)					Rain-fed rice (35)				Vegetable (18)
AEZ-7	53–59		Irrigated rice (15)				Jute (12)				Rain-fed rice (20)			
AEZ-8	60–65			Irrigated rice (20)						Rain-fed rice (20)				
AEZ-9	56–66		Fallow	Irrigated rice (15)					Rain-fed rice (41)					Fallow
AEZ-10	65–78			Irrigated rice (40)					Rain-fed rice (40)					
AEZ-11	118–130		Vegetable (25)		Irrigated rice (40)					Rain-fed rice (45)				
AEZ-12	16–20		Wheat		Irrigated rice (20)				Jute (20)				Wheat (20)	
AEZ-13	3–6		Irrigated rice (20)						Rain-fed rice (20)					
AEZ-14	16–23	1	Irrigated rice (20)			fallow								Irrigated rice (20)
		2	Irrigated rice (20)			Jute (12)								
AEZ-15	13–23		Irrigated rice					Rain-fed Rice						
AEZ-16	30–43													
AEZ-17	16–23	1	Irrigated rice (29)					Rain-fed rice (49)				Mustard (16)		
		2	Irrigated rice (29)					Rain-fed rice (49)						
AEZ-18	3–7		Irrigated rice (40)			Soybean (19)				Rain-fed rice (43)				Irrigated rice (40)
AEZ-19	7–13	1	Irrigated rice (23)			Fallow								
		2	Irrigated rice (23)				Jute (20)							
AEZ-20	33–40		Vegetable (15)						Rain-fed rice (34)					Vegetable (15)
AEZ-21	50–66		Fallow		Irrigated rice (38)				Fallow					
AEZ-22	82–90		Irrigated rice (20)					Rain-fed rice (20)						Irrigated rice (20)
AEZ-23	23–33			Irrigated rice (15)						Rain-fed rice (23)				
AEZ-24	7–13							Rain-fed rice (10)						
AEZ-25	59–66	1	Potato/mastered (14)	Irrigated rice (20)									Potato/mastered (14)	

### 3.2 Respondents’ characteristics

#### 3.2.1 Demographic profiles of participants

The respondents to the survey included 865 farmers from all 30 AEZs, with a significant majority (96.2%) being male and the remaining (3.8%) female. The age distribution revealed that none of the surveyed farmers were younger than 16 years. Among the participants, 30.74% were between 16 and 35, 66.82% were between 36 and 70, and just over two percent (2.44%) were 71 and older (Table 3).

**TABLE 3:** Characteristics of farmers’ status, weeding strategies, and sources of irrigated water.

Factor	Categories	Number (Frequency)
Gender	Male	832 (96.2%)
	Female	33 (3.8%)
Age (Years)	16–35	266 (30.74%)
	36–70	578 (66.82%)
	Above 70	21 (2.44%)
Education	No literacy	320 (36.99%)
	Primary (Class 1–5)	200 (23.06%)
	Secondary (Class 6–10)	238 (27.5%)
	Higher Secondary (Class 11–12)	74 (8.63%)
	Graduate	33 (3.82%)
Rice cultivation experience (years)	Less than 5	105(12.16%)
	5–10	112 (12.98%)
	11–15	108 (12.51%)
	16–20	158 (18.25%)
	21–25	88 (10.18%)
	26–30	88 (10.14%)
	Above 30	203 (23.51%)
Total cultivation area (acres)	0.33–0.79	251 (41.8%)
	0.80–1.29	219 (26.1%)
	1.30–1.99	171 (20.4%)
	2.00–2.64	40 (4.8%)
	2.65–3.65	34 (4.1%)
	3.66–10	24 (2.9%)
Weed management strategies	Herbicide-based weeding	(24) 2.7%
	Use herbicide and manual weeding	(686) 79.3%
	Manual weeding	(155) 17.8%
Sources of irrigation water	Groundwater	770 (89%)
	Surface water	69 (8%)
	Others	26 (3%)

#### 3.2.2 Education levels

Virtually all participating farmers disclosed their educational backgrounds. Of these, 36.99% were illiterate, 23.06% had completed primary school (≤5 years of schooling), 27.50% had finished secondary education, and 8.63% had attained higher secondary education. In contrast, a smaller percentage (3.82%) had received tertiary education.

#### 3.2.3 Experience in rice cultivation

A significant number of farmers (23.5%) reported having more than 30 years of experience in rice cultivation. Around 18.2% of farmers have 16–20 years of experience, while approximately 13% and 12.5% have 5–10 years and 11–15 years of experience, respectively. Only slightly more than 10% of farmers mentioned having 21–25 years and 26–30 years of experience in rice cultivation. Approximately 12.2% of farmers are new to rice farming, with less than 5 years of experience.

#### 3.2.4 Land ownership and distribution

The study included farms with a minimum of 0.33 acres of rice-growing land. The farmers surveyed cultivate rice on land ranging from 0.33 to more than 10 acres. Most (41.8%) farmers cultivate between 0.33 and 0.79 acres. Around 60.1% of all surveyed farmers own less than one acre of land for regular crop cultivation. Additionally, 26.1% and 20.4% of farmers cultivate land ranging from 0.8 to 1.29 acres and 1.3 to less than 2 acres, respectively. Furthermore, 4.8% and 4.1% of farmers cultivate land between 2 to 2.64 acres and 2.65–3.65 acres, respectively. Only 2.9% of farmers cultivate land spanning from 3.66 to 10 acres for rice production. The average land size based on the countrywide data is 1.2 acres.

#### 3.2.5 Frequency of land utilization for crop production

Interesting trends were noted in the findings about land allocation for crop production. According to the data, 45.18% of farmers utilize their land for two crop cycles annually, while 35.08% allocate their land for three crop cycles. Surprisingly, 19.74% of farmers focus on a single crop cycle. Within the three-crop-cycle areas, it was observed that the cultivation of two rice crops is predominant (80%), while the practice of three rice crops in succession is rare. All participating farmers grow transplanted rice. The survey focused on rice cultivation in Bangladesh during two main seasons: *boro*, which runs from January to May and involves irrigation, and *aman*, which typically takes place from July to November and relies on rain, with occasional irrigation if there is not enough rain. In some AEZs (e.g., AEZ-19), rice cultivation is not feasible in the *aman* season in most places due to submergence (low-lying land).

#### 3.2.6 Rice varieties

Most rice growers cultivate high-yielding varieties (90%). The shares of low-yielding local varieties and landraces are relatively small. The cultivation of local varieties is confined mainly to the *aman* season. Among high-yield, open-pollinated varieties dominate the field. F1 hybrids have only a small share.

### 3.3 Weed management practices and water use

#### 3.3.1 Herbicide application and manual weeding

**FIGURE 1:**
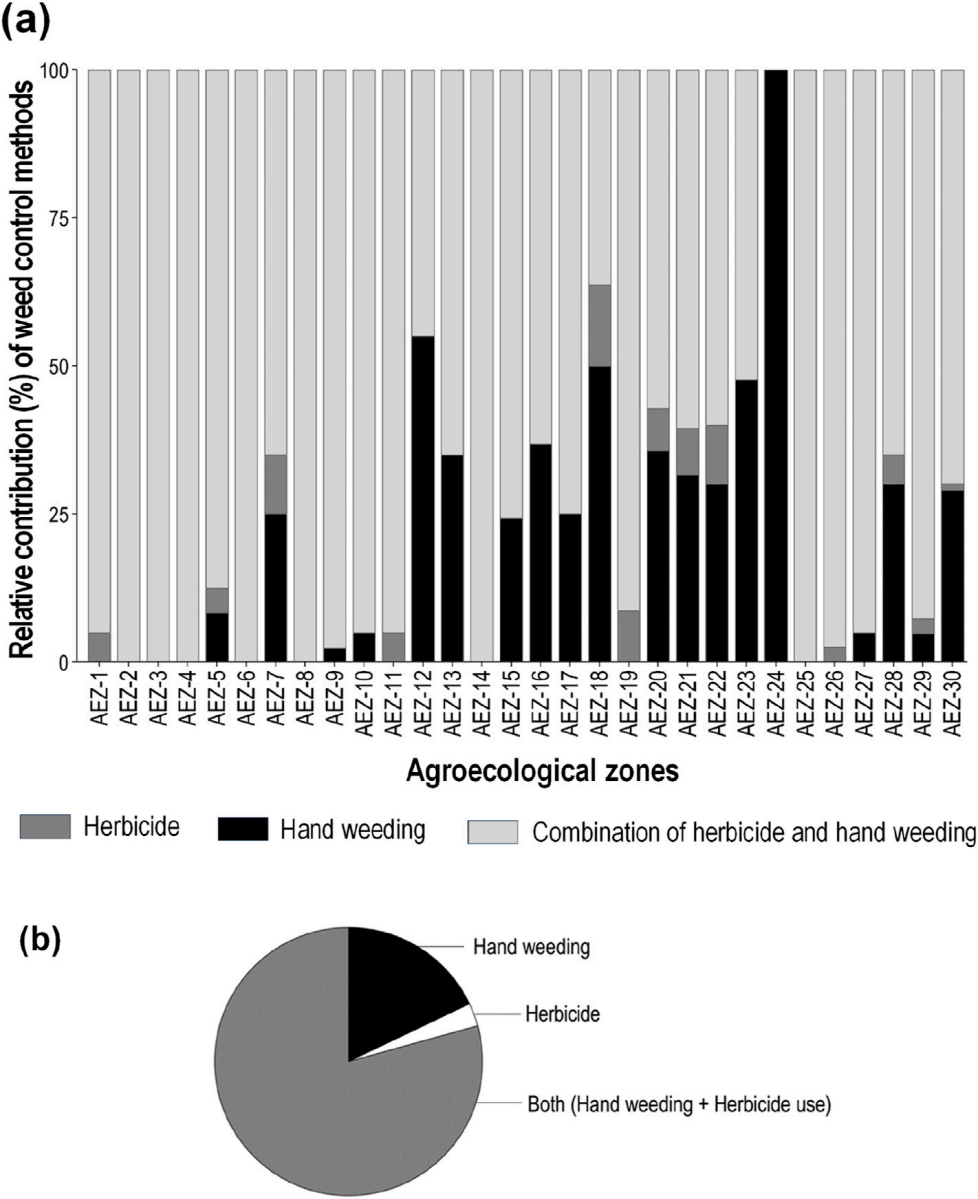
**(A)** Zone-wise relative contribution of various weed control methods: hand weeding, herbicide-based weeding, and their combinations. Each bar represents a specified agroecological zone of Bangladesh. **(B)** Overall contribution of major weed management practices. Data represent the average of all AEZs in Bangladesh.

As a traditional practice, all surveyed farmers believe that manual hand weeding is the best method of weed control, if possible. However, only 17.8% did not apply herbicides and solely depended on manual hand weeding. The main reason is the high cost and availability of labor. Our data suggest that 79.3% of the respondents use a combination of manual weeding and herbicides. Only a small proportion of farmers, 2.7%, used herbicides solely. The average weed control strategies by zone are illustrated in Figure 1. The frequency of people using herbicide increases, and the frequency of people using hand weeding tends to decrease slightly, and *vice versa*, as there is a small significant relationship between the variables (Supplementary Table S1).

**FIGURE 2:**
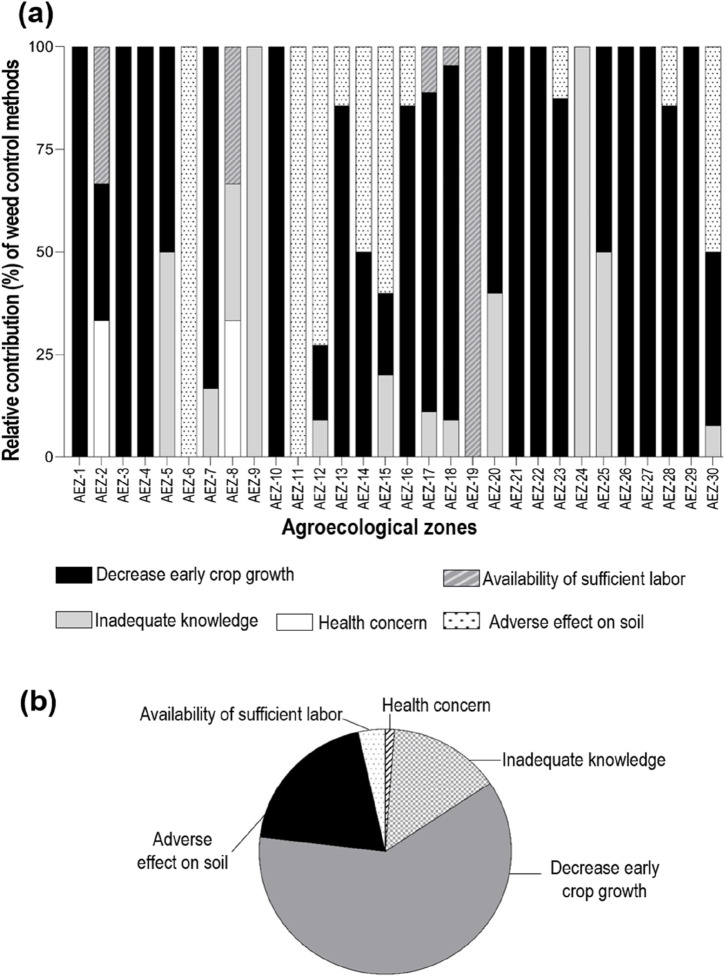
Reasons for not adopting herbicides (zero-herbicide cultivation). **(A)** Response variation in 30 AEZs and **(B)** the country’s average data. Only the perceptions of farmers relying solely on manual weeding without any herbicides are considered.

AEZ-18 and AEZ-22 possess the highest percentage of solely herbicide adopters, and AEZ-12 had the smallest proportion of herbicide users, except for AEZ-24, where no farmers reported using herbicides. They showed no interest in herbicides even after learning about the benefits. Due to its small agricultural area and traditional farming methods deeply ingrained in the community, farmers may favor manual weed control techniques over chemical inputs. In addition to the high labor cost and availability, the reasons for not using herbicides differed across AEZs (Figure 2). Approximately 14.2% of farmers do not have sufficient knowledge of the efficacy of herbicides. Approximately 81.3% of the non-adopters believed that herbicide use causes harm to crops or soil, and manual weeding is more beneficial to yield. Very few farmers (1.3%) think herbicides may harm their health. Approximately 3.2% of farmers know about the efficacy of the method but do not use it as they have a sufficient workforce to conduct manual weeding. Most non-herbicide users (80%) expressed their willingness to accept herbicides, provided that they will not damage crops or soil. Interestingly, in AEZ-18 and AEZ-12, although farmers know about herbicides, they do not use them. They use collected weed as fodder. Weeds are a kind of minor crop to them.

Thus, inadequate knowledge of efficacy appears to be the main reason for not adopting herbicides. At the same time, marginal farmers with an adequate workforce in their families also feel that herbicide is not the most profitable option. Statistical analysis showed a non-significant negative correlation (small relationship) between land size and herbicide adoption. This indicates that herbicide adoption is random (Supplementary Table S2).

#### 3.3.2 Timing and frequency of herbicide application

Respondents from all AEZs apply herbicides within 1–7 days of transplantation. This practice effectively reduces the emergence of weeds in muddy fields. However, in some cases, all weeds are not entirely suppressed. Thus, weeds need to be removed again within 3–6 weeks. Rice is not tolerant to most of the post-emergence systemic herbicides. Hence, manual hand weeding is essential. Approximately 44.3% of farmers must conduct one manual weeding. On the other hand, two hand-weeding sessions is the most common requirement, and around 47.4% of farmers have used this practice in their fields.

According to farmers, the presence of high amounts of weed propagules (8%), lack of standing water after transplantation (80%), and improper doses of herbicides (10%) are the primary cause of the weed emergence and subsequent requirements of manual weeding even after using herbicides.

#### 3.3.3 Frequency and timing of manual hand weeding

Apart from herbicide treatment, most farmers (97.3%) used hand weeding alone or in combination with herbicide application. This method included either a single session of hand weeding or a combination of hand weeding plus herbicide application. Farmers who rely solely on manual weeding typically conduct one to three sessions of manual weeding to remove unwanted plants by hand, ensuring the optimal growth of their crops. However, the weeding frequency varied by AEZ.

For those who do not adopt herbicides, 32% opt for a single manual weeding session, 57% for two sessions, and 10.9% for three sessions. On the other hand, herbicide adopters reported that manual weeding continues to be an essential part of the process despite using herbicides. In those cases, the frequency of hand weeding was reasonably balanced, with 45.2% of farmers doing one session and 48.4% doing two sessions, while the requirement of three sessions is rare (6.4%).

The farmers expressed that the frequency of manual weeding was contingent upon the prevalence of weeds in the rice field. A significant number (49.3%) of farmers weeded by hand 15 days after planting, followed by a second session after 30 days. Various timeframes for hand weeding were observed within and across AEZs, with no indication of hand weeding being necessary beyond 7 weeks.

#### 3.3.4 Types-pre-emergence and post-emergence

In some AEZs, rice fields are overrun by weeds. Therefore, pre-planting weed clearing is necessary. In those cases, non-selective post-emergence herbicides are used. Glyphosate and paraquat are the primary herbicides, contributing 33% and 67%, respectively. Such pre-cleaning also varies by season and AEZs. It may be required in *aman* season in some AEZs, while necessary in *boro* season elsewhere.

Farmers’ perceptions of herbicide effectiveness were focused on the pre-emerging stage, with 81.9% reporting herbicide application at this time. In contrast, only 6.6% of farmers used herbicides during the post-emerging period. Among the herbicide users, 92.7% of respondents used pre-emergence-type herbicides. This class of herbicides is mainly applied within 1–7 days of the transplantation of rice seedlings. A total of 86% of farmers apply herbicide by this time. Such an application prevents the emergence of weeds. By contrast, herbicides are used after weed emergence to a much lesser extent (7.3%) in some cases. We observed such herbicide application (after weed emergence) exclusively in AEZ-19 and AEZ-29.

#### 3.3.5 Active ingredient

**FIGURE 3:**
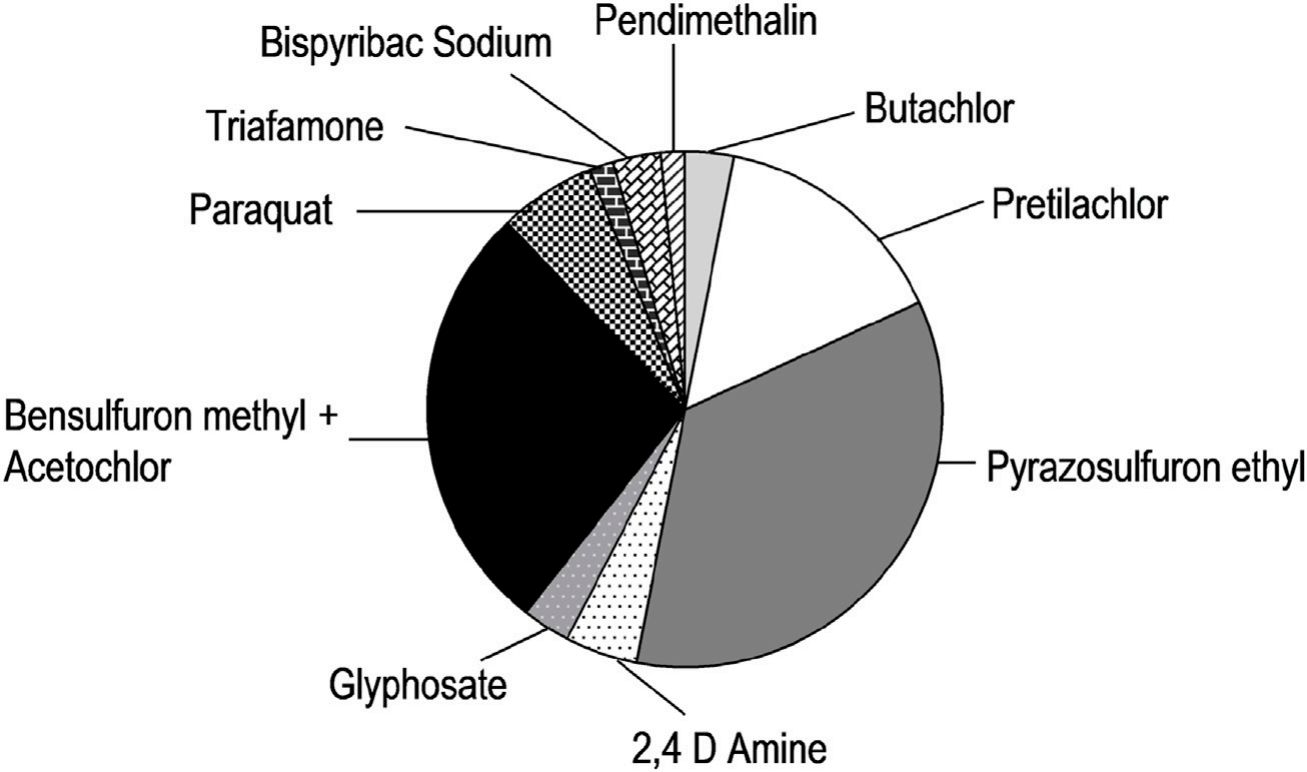
Pie chart showing various active ingredients of herbicides used by the farmers in all 30 AEZs.

**FIGURE 4:**
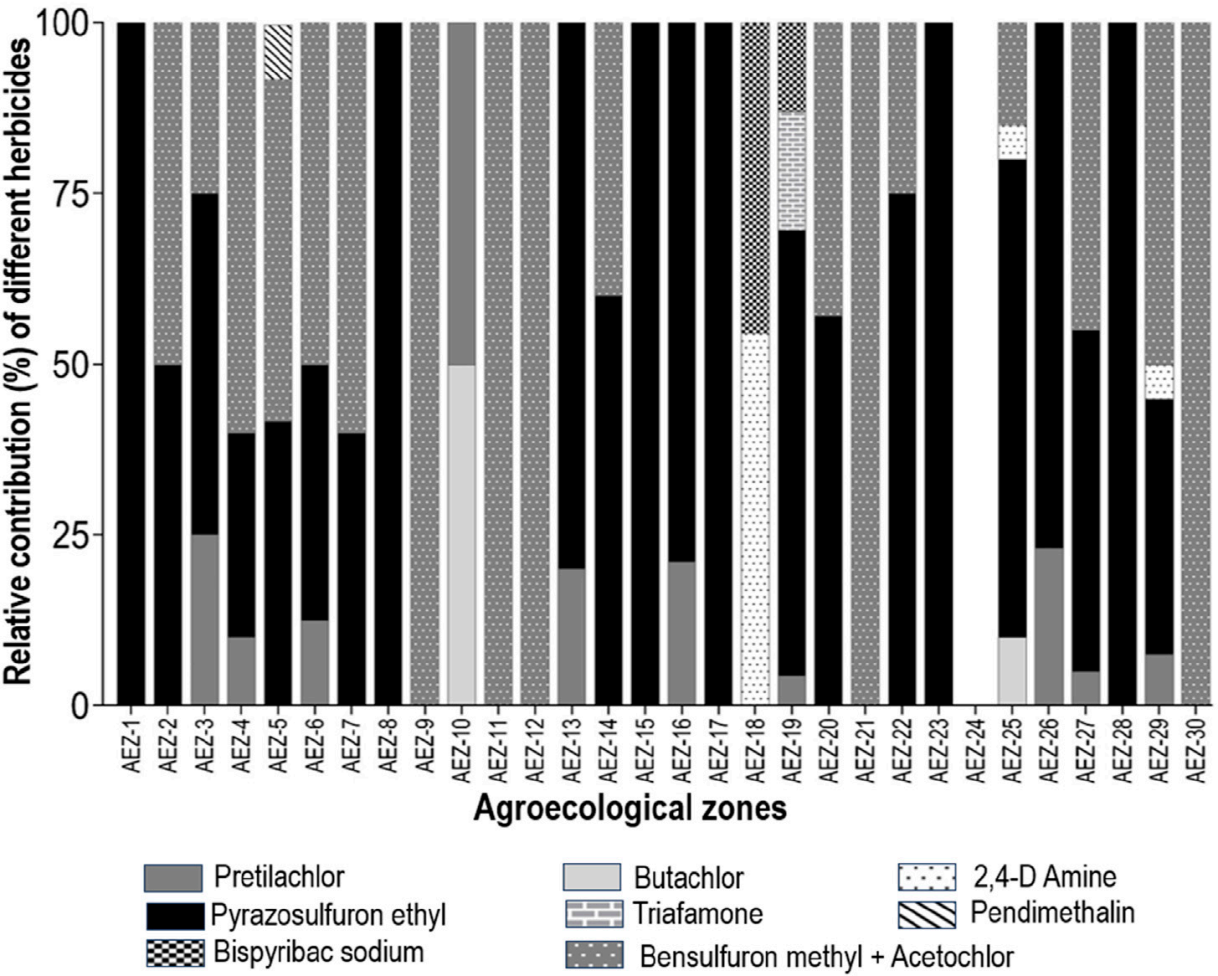
Percentage of various herbicides’ active ingredients used in the 30 different AEZs. Each bar represents a specified AEZ.

We noticed farmers across the country use 10 formulations to control weeds. Use of glyphosate and paraquat is limited to pre-cultivation weed control. Various formulations are used after seedling transplantation. Among the various herbicides enlisted in this survey, pyrazosulfuron ethyl emerged as the most frequently used herbicidal compound, with approximately 35% of farmers using it. The combination of bensulfuron methyl (4%) and acetochlor (14%) ranked next at around 27%, followed by pretilachlor, which accounted for 15%. Some other chemicals, such as paraquat, glyphosate, 2,4-D amine, butachlor, triafamone, and bispyribac sodium, are used by farmers all across the AEZs. The relative contribution of these chemicals is shown in Figure 3. The utilization of different herbicides in 30 AEZs is shown in Figure 4. Only 0.5% of farmers are conscious of the active ingredients. Most know herbicides and their effectiveness by trade name.

### 3.4 Availability of herbicides in the marketplaces

We collected data from agrochemical shops in all 30 AEZs during the survey. A total of 49 trade-named herbicides were listed. These products represent ten different herbicide chemicals (Table 4). Among them, eight products contained single-active ingredients, while two were combined formulations. In all cases, shopkeepers report that most farmers seek their advice on selecting appropriate herbicides. Shopkeepers received training on herbicide usage from their manufacturer/supplier companies.

**TABLE 4:** List of herbicides used by farmers in studied locations classified using the WHO Hazard Class and Health Effects, 2019 (World Health Organization, 2020).

Number of products	Active ingredients	Mode of action	WHO Class	Agroecological zone
2	Butachlor	Pre-emergence	Class III	AEZ-25, 10
10	Pretilachlor	Pre-emergence	Class III	AEZ-19, 29, 13, 10, 3, 27, 6, 4, 16, 26
8	Pyrazosulfuron ethyl	Pre-emergence	U	AEZ-25, 19, 14, 29, 23, 5, 28, 22, 20, 13, 17, 2, 1, 3, 27, 6, 8, 7, 4, 15, 16, 26
3	2,4 D Amine	Pre-emergence	Class II	AEZ-25, 29, 18
4	Glyphosate	Post-emergence	Class III	AEZ-14, 29
4	Paraquat	Post-emergence	Class II	AEZ-14, 29, 28, 6
12	Bensulfuron methyl+ Acetochlor	Pre-emergence	U Class III	AEZ-25, 14, 29, 5, 22, 20, 11, 2, 3, 27, 6, 12, 7, 4, 9, 21, 30
2	Triafamone	Pre-emergence	Not listed	AEZ-19
2	Bispyribac sodium 40%	Pre-emergence	Class III	AEZ-19, 18
2	Pendimethalin	Pre-emergence	Class II	AEZ-05

Glyphosate and paraquat were the most common herbicides used when pre-cultivation weed cleaning is required. These two herbicides were sold under eight different trade names and contributed 3% and 6%, respectively. Compared to pre-emergence rice herbicides, glyphosate and paraquat were sold in much higher quantities in AEZ-29. Shops in AEZ-24 did not sell any herbicides.

Among the pre-emergence herbicides, bensulfuron methyl with acetochlor is sold under 12 different trade names. Pyrazosulfuron ethyl was sold under eight trade names, pretilachlor under 10 trade names, and 2,4 D amine under three trade names. The price of herbicides is not influenced by AEZs but rather by the brand of the herbicide. Paraquat is the cheapest at $1 per acre among post-emergence types. In contrast, triafamone, pyrazosulfuron ethyl, and bensulfuron methyl with acetochlor were sold at $2.08 per acre, $0.80 per acre, and $1 per acre, respectively. According to the labels, all product is registered.

### 3.5 Cost, yield, and profitability

#### 3.5.1 Cost of weed control

**FIGURE 5:**
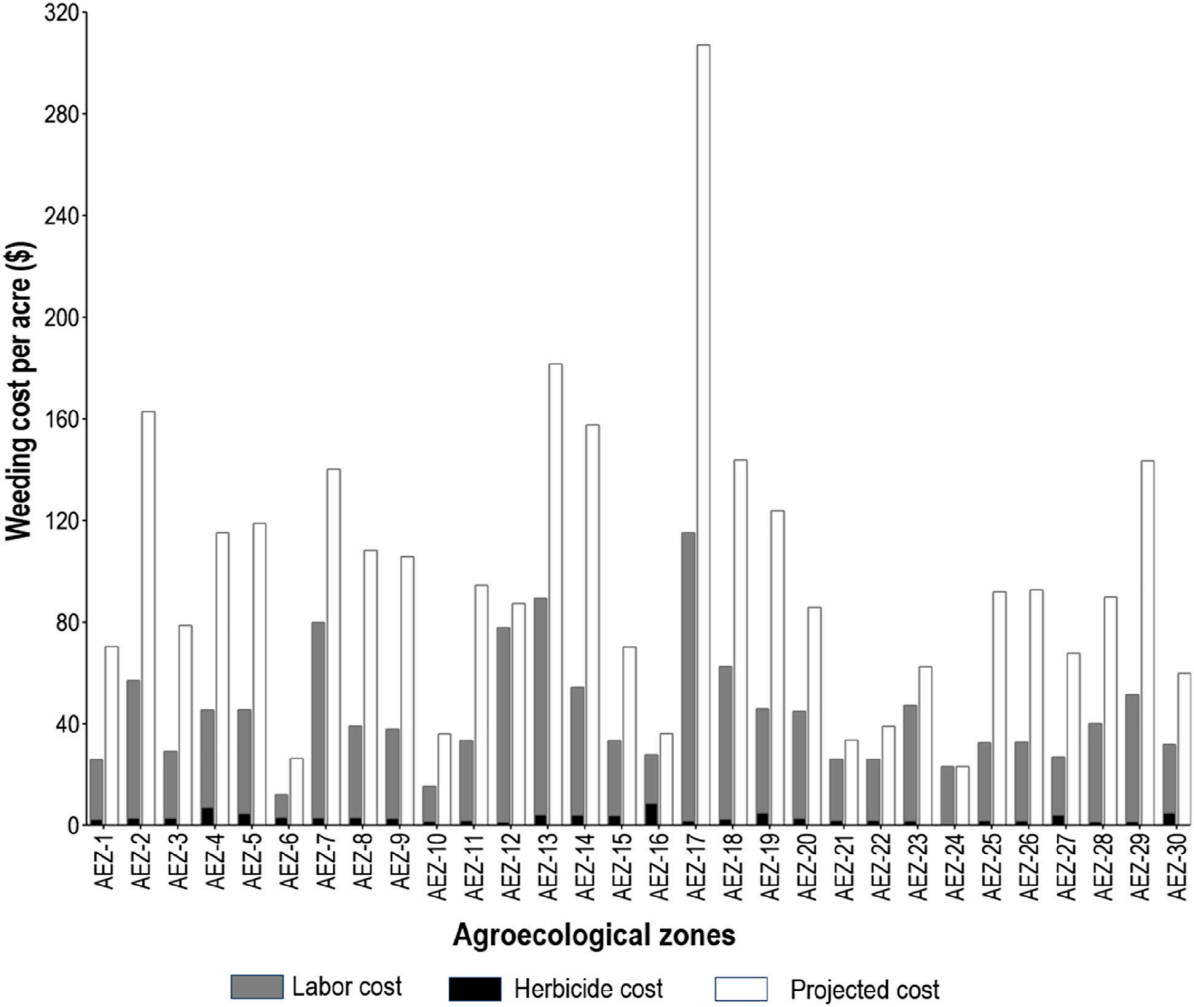
Differences in weeding cost. The cost of the currently used combination of herbicide and manual weeding method is compared to the no-herbicide scenario (projected cost based on a full hand weeding session).

We gathered data on weed control expenditures and categorized them as herbicide costs and manual weeding expenses. Total cost varied by AEZs, ranging from $12–$116 during the entire crop life. Where herbicides are used solely, the expenditure is the least. Herbicide costs at retail shops ranged from $1–$3 for each acre of land, depending on brand and active ingredients. In most regions, using a combination of herbicide application and manual weeding costs less than relying on manual weeding alone (Figure 5). Such a difference in expenditure is associated with high labor wages. During a narrow 3-week window in a region, labor shortages for weed control often lead to price increases. Labor wages ranged from $3.2 to $7 per day during the survey period. In addition to herbicides, labor cost for weeding is an average of $43.7 for each acre. A significant difference (*p* < 0.05) in total weed management costs was observed among the 30 AEZs, as determined by a one-way ANOVA test. During the survey, we aimed to assess the role of herbicides in weed control expenditure and profitability of rice cultivation. We requested farmers to estimate the cost of weed control through manual labor if herbicides were eliminated, compared to the current method of using both herbicides and manual labor. Farmers projected that if no herbicides are used, the average cost for weed management would be $98.5 per acre (Supplementary Table S5). Responses suggested that in AEZ-17, the weed management cost would be as high as $307 per acre. The savings due to herbicide application would be as high as 65%. In conclusion, rice cultivation would not yield significant profits in most AEZs if manual hand weeding was the sole method employed. A paired sample *t*-test was performed to determine the effect size of herbicide adoption in reducing the cost, and significant differences were observed with a large effect size (Supplementary Table S3).

#### 3.5.2 Negative effects of herbicides and mitigation measure

Regarding the perceived effects of herbicide use, 44.5% of farmers reported that herbicides harmed the growth of rice seedlings. These include reduced growth and sluggish tiller formation. Association between herbicide application and consequent adverse effects are found to be statistically significant with a large relationship (*p* < 0.001). Approximately 35% of farmers apply additional fertilizers to compensate for the early growth cease of seedlings. Most use urea and sulfur, while a few percent use their combinations. Some farmers (10%) were willing to apply additional fertilizer but were held back by the hefty cost.

#### 3.5.3 Yield observation

Most farmers (64.1%) agreed that using herbicides significantly increased their yield. They immediately attributed the increase in production to their agronomic practices, which included herbicides. They asserted that higher yields do not directly result from the herbicide application; however, they can correlate. This subset of farmers noticed that manual weeding techniques could yield results similar to those obtained with herbicides, eliminating the need to use them to increase yields directly. However, timely weeding is not often possible when it depends on labor forces, negatively affecting yield. Herbicide application, by contrast, requires little labor and is easily manageable. Notably, most farmers agreed that herbicide-based weed control was the least costly. Most respondents consented that this cost-saving feature was a positive consequence. Approximately 34.9% of the farmers disagreed with herbicide-mediated yield increase. They mainly reasoned the adverse effects of herbicides on crop growth. Only 1% of the farmers who participated in the survey had no opinion about the impact of herbicide treatment on crop growth. These farmers inferred that there was no apparent connection between the usage of herbicides and increased crop yields. Statistical analysis, however, suggests a significant association with a medium positive relationship has been found in the chi-square test (Supplementary Table S1). Thus, herbicides indirectly contribute to yield increase and benefit overall rice production in the country.

### 3.6 Knowledge of farmers, health, and environment

#### 3.6.1 Knowledge and attitude

Our questionnaires also assessed the farmers’ knowledge of herbicide ingredients, dose, and safety considerations. We found that only 5.1% of the farmers read labels to consider application dosage, appropriateness, and safety. The chi-square test suggested that the number of people aware of these issues is not concentrated to any AEZ but is somewhat randomly distributed.

We observe a moderate degree of positive association between herbicide adoption and the education qualification of the participants (Supplementary Table S1). Most farmers (69.3%) depend on pesticide shops to select herbicides and their dosages. Approximately 15.5% of farmers learned appropriate herbicide formulations as trade names from the experience and suggestions from their friends and neighbors. According to farmers, suggestions from government extension field workers are rare (8%). Almost no farmers are aware of the active ingredients of the herbicides.

Approximately 72.7% of farmers reported that applying herbicides did not cause any harmful effects on their health. The association of the variables is found to be non-significant (Supplementary Table S1). They observe this in contrast to insecticide applications. A small proportion of farmers reported minor health issues such as headaches and eye irritation. Interestingly, approximately 15.4% of farmers think that herbicides may harm the ecosystem, although this percentage is statistically not significant (Supplementary Table S1). No farmers see any immediate adverse effects on the animals, including frogs, earthworms, fish, or other beneficial animals inhabiting the rice field.

Only a small number of respondents (12.4%) have linked the use of herbicides to adverse health effects. Wearing personal protection equipment is, therefore, rare. Nevertheless, they admit that using PPE would be better. The use of personal protective equipment (PPE) during herbicide application was cited as a safety practice by 32.2% of respondents. Despite the potential risks they assume, 67.8% of respondents admit to not using essential personal protective equipment (PPE). Notably, a considerable segment (78%) recognized the necessity of PPE but acknowledged their non-adherence to this safety measure. As mentioned earlier, most farmers depend on shopkeepers’ recommendations regarding efficacy and safety.

#### 3.6.2 Knowledge of herbicide toxicity

Within the herbicide-using group, 34.6% of farmers reported adopting herbicides for more than a decade. A significant proportion (63.2%) of this subgroup cited the adverse effects on the development of rice seedlings as the main reason for their unwillingness to use herbicides. On the other hand, 36% did not use herbicides because they knew enough about alternatives. Only 1.3% of respondents said health issues prevented them from using herbicides. Around 18.1% of farmers mentioned that applying herbicides may harm soils. Regarding the method, 60.1% of farmers chose to combine herbicides with fertilizers, while 24.5% preferred direct spraying. A small number of farmers used both methods.

#### 3.6.3 Inefficacy of herbicides

In almost all AEZs, farmers complained about the efficacy of herbicides on several weed species. Many weeds can skip the pre-emergence treatment during rice cultivation. A list of such weeds is provided in the Supplementary Figure S1. These weeds necessitate manual hand weeding, adding extra financial burden on farmers. Thus, effective post-emergence herbicides would be helpful.

### 3.7 Farmer needs and response to future intervention

Farmers urge for appropriate technology to control some weeds that are not easily controlled by currently used pre-emergence herbicides. Notably, post-emergence herbicides are even more harmful to rice crops. Therefore, their relative efficacy to weed species and stages should be studied to determine the effective use of herbicides and minimize their effects. Most current herbicides are ineffective during the post-emergent phase, which poses a significant problem. Application is, therefore, not flexible. Rice can be genetically engineered to resist herbicides, including those of post-emergence types. Among the surveyed farms, only 1% of the farmers know about GMOs or genetically engineered crops. At this point, they have yet to learn about the safety or efficacy of GE traits. However, 99% are willing to cultivate GE rice varieties if they better tolerate herbicides, are profitable, and contribute to a higher yield.

As mentioned earlier, the efficacy of pre-emergence herbicides requires standing water after their application. We asked farmers about possibly introducing new rice varieties that are tolerant to post-emergence herbicides. It appears that farmers often practice AWD methods for rice cultivation. They observe that the AWD method benefits from water savings and cost reduction. However, it is not practiced much as a consequent emergence of numerous weeds. If post-emergence herbicide-tolerant varieties are available, they want to apply AWD and save water.

### 3.8 Response pattern on survey questions

**FIGURE 6:**
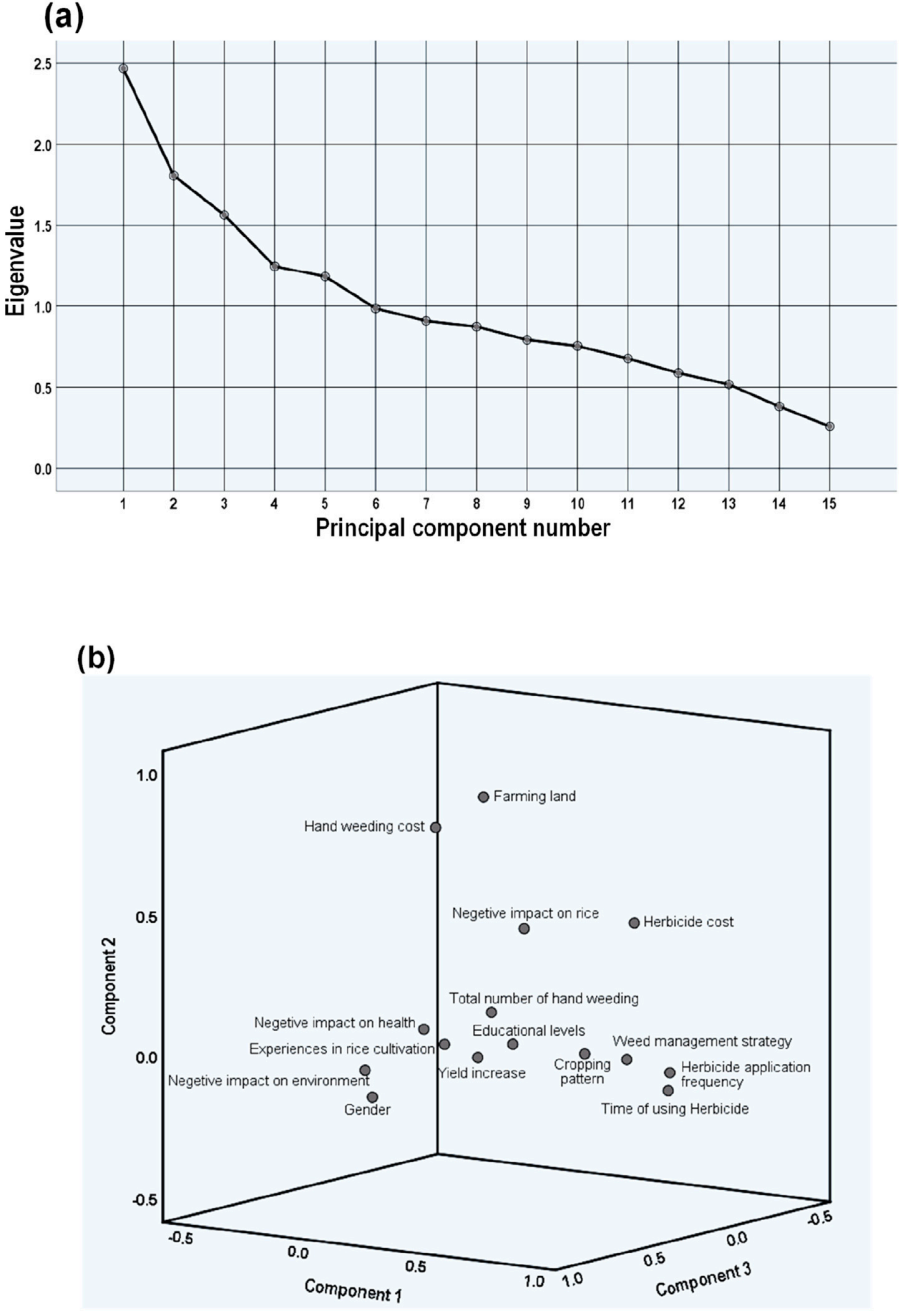
Factor loadings of farmers’ questionnaire items on herbicide use. **(A)** A scree plot representing the eigenvalues and the proportion of variance accounted for by the principal components and **(B)** a component plot shows the factor loading of the 3D model after varimax rotation.

The latent structure of the weed management strategy (WMS) was examined using principal component analysis with varimax rotation. The scree plot selects the number of components based on the eigenvalues. It arranges the eigenvalues from the largest to the smallest. In our data, the scree plot determines the optimal number of components to be considered in the model (Figure 6A). The initial analysis revealed five factors with eigenvalues larger than 1, explaining 55.12% of the variance (Supplementary Table S6). The eigenvalue of each component in the initial analysis is plotted. The components on the shallow slope contribute little to the solution. Our data exhibit a steep curve pattern, followed by a bend at the fifth component (elbow threshold), and then nearly form a straight line. Such a pattern suggests a potential five-factor solution for the WMS.

Figure 6B illustrates the loading of the five factors for the individual items in the questionnaire. Questionnaire items that have high loadings on factor 1 (only) are, for example, “frequency of herbicide application in the field,” “time to use herbicide,” and “herbicides cost in the field;” items that have high loadings on factor 2 (only) include “cultivated farming areas” and “hand weeding cost.” The other nine items are allocated as follows: “negative impact on the environment,” “gender,” “number of crops grown in a field around the year,” and “negative impact on health” have a high impact on factor 3, “herbicide on the yield increase,” and “hand weeding number” have an impact on factor 4, and “experiences in rice cultivation,” “participants’ education qualification,” and “negative impact of herbicide” have an impact on factor 5. Items have high loadings on other factors. Therefore, this indicated that factor 1 largely summarized the properties of the WMS.

### 3.9 Relationships among various factors

The multinomial logistic regression analysis was conducted to examine the effects of cost and herbicide impact on farmers’ weed management practices. The key results are summarized in the Supplementary Table S4. The overall fit of the model was evaluated using the likelihood ratio chi-square test, Pearson and deviance chi-square tests, Cox and Snell pseudo-R-square, and Nagelkerke pseudo-R-square. The likelihood ratio chi-square test was statistically significant (*p < 0.0001*). This suggests that our model containing the full set of predictors fits the data significantly better than the intercept-only model. The Pearson chi-square and deviance chi-square tests were both statistically non-significant (*p > 0.05*), suggesting the robustness of the model. High pseudo-R-square values of Cox and Snell (0.665) and Nagelkerke (0.958) indicate that the predictors in the model explain a substantial portion of the variance in the dependent variable.

From the multinomial regression table (Supplementary Table S4), we see that higher herbicide costs do not influence the probability of choosing hand-weeding alone (O.R. = 0.993) or a combination of hand-weeding and herbicide (O.R. = 0.993) compared to herbicide use only. More frequent herbicide use decreases the probability of choosing hand-weeding (O.R. = 3.209E-07) and a hand weeding–herbicide combination (O.R. = 0.014). This shows that farmers perceive more frequent applications of herbicides as saving costs. On the other hand, the current tendency of hand-weeding increases the likelihood of choosing hand-weeding alone (O.R. = 1102.775) and a combination of hand-weeding and herbicide use (O.R. = 2542.574). This indicates that farmers would preferably depend on only manual labor if it were available, and the cost was competitive to herbicides.

In addition to costs, potential negative effects on early seedling growth are important determinants of farmers’ choice of weeding method. Farmers who perceive herbicides as negative for rice are less likely to support herbicides alone (O.R. = 0.003). The impact of herbicide use on the environment and whether there is a perceived yield benefit to using herbicides influence the model. However, they are not statistically significant. Overall, the results suggest that economic factors and seedling health are key factors that influence the adoption of weed management strategies.

## 4 Discussion

Farmer surveys that evaluate the current status and determine their knowledge and perception of weed problems, their limitations in dealing with the situation, and their attitudes in the weed management system are one of the most vital approaches for data assembly. Therefore, we focused on the current knowledge level of the farmers regarding weed control, as well as their perceptions and attitudes toward the efficacy and safety of herbicide usage. Our survey covers the whole of Bangladesh, which is represented by 30 AEZs. It provides valuable insights into farmers’ variable weed management practices, their characteristics, attitudes, the challenges they face, and possible interventions.

Due to the edaphic and climatic diversity of cultivation areas (AEZs), weeds are also diverse. Their control measures require different practices. Our study mainly found and focused on transplanted rice in both dry and rainy seasons. The demographic profile of surveyed farmers indicates a male-dominated workforce, with a significant portion (66.82%) of respondents between 36 and 70 years old, indicating a mature farming population. Most farmers have substantial experience in rice cultivation (over 50% with more than 15 years).

Our research indicates that a significant percentage of farmers (37%) does not have formal education, underscoring a potential need for educational interventions and assistance to improve agricultural practices and productivity. A noticeable percentage of newcomers to rice farming was observed. Age and experience distribution potentially highlights knowledge transfer to younger generations, ensuring sustainable rice production in the country. This also translates into well-established weed management approaches.

Most farmers cultivate relatively small landholdings, with less than one acre owned by most respondents. This highlights the prevalence of small-scale farming. Despite variations in land size, the average acreage remains modest, suggesting limited land consolidation. According to World Bank data, the current average arable land size is 0.12 acres *per capita*. Thus, our survey data are consistent with the national average. The frequency of land utilization for crop production varies, with a significant proportion of farmers opting for two or three crop cycles annually. Transplanted rice is the primary crop, predominantly cultivated during the *boro* and *aman* seasons.

Weed control methods varied by AEZs as regional factors, such as the availability of labor, farmers’ economic status, etc., were diverse. Sole use of herbicides or manual labor is not the ideal case. A significant proportion of farmers deploy only manual weeding. Such a limited adoption of herbicides among farmers, especially smallholders, is due to various factors, including high costs, lack of knowledge about herbicide application, readily available workforce from their own family, and unwillingness to use toxic agrochemicals. Market surveys suggest that access to modern agricultural technologies and herbicides is not the predominant factor. This is in contrast to earlier findings (Rahman et al., 2019).

A significant number of this group perceived that herbicides negatively affect early crop growth. Farmers’ observations align with scientific investigations. A study from Zhang (2015) found that although bensulfuron methyl and pyrazosulfuron ethyl are good herbicides for controlling weeds, they can stunt the growth of rice seedlings and reduce overall crop vigor. It has also been found that these herbicides can cause phytotoxic effects on rice plants, resulting in delayed development and stunted growth. (Chauhan, 2013; Zhang et al., 2015).

Labor-intensive manual methods may be effective on a small scale but can be time-consuming and costly, posing challenges to farmers during peak seasons. Farmers’ unwillingness to use herbicides in AEZ-24, attributed to factors like limited access to herbicides and traditional farming methods, highlights the need for tailored interventions to address specific regional challenges.

The sole use of herbicides is limited in certain places where the weed predominance is the lowest. At the same time, many such farmers admit that they cannot afford the manual labor that is needed. A combination of herbicide use and manual labor is the most common scenario. This indicates a growing acceptance of herbicides alongside traditional practices. At the same time, it is also clear that many weeds are not eliminated by herbicide treatments. The requirement for manual labor following herbicide treatment varied. This is due to several factors, such as the high presence of weeds, lack of knowledge on proper dosing, and unavailability of standing water after transplanting. This highlights the adverse effects of herbicides and potential herbicide-resistant weeds that need further attention.

Our data also revealed a preference for pre-emergence herbicide application. This aligns with the survey findings on weed control practices, highlighting the focus on preventing the initial emergence of weeds. Most farmers (86%) apply herbicides within 1–7 days of transplanting rice. Manual weeding alone or in combination is needed within 3–6 weeks of transplantation. This suggests a very high demand for a manual workforce within a limited period in a region. This eventually makes weed removal too tricky for many farmers, impacting productivity.

Glyphosate and paraquat, two post-emergence herbicides, are used by farmers to prepare their land before rice planting in certain zones, such as AEZ-19, where land is left fallow for a few months. Consequently, weed prevalence is high, and pre-planting land cleaning is essential. Therefore, these chemicals are not directly used for weed management during rice cultivation but are crucial for pre-cultivation weed control. Various formulations of chemicals are employed during cultivation for weeding purposes. Pyrazosulfuron ethyl emerges as the most frequently used herbicidal compound, followed by other chemicals such as bensulfuron methyl with acetochlor and pretilachlor. Interestingly, the data indicate limited awareness among farmers regarding the active ingredients of herbicides, with most farmers identifying herbicides by trade names rather than active ingredients.

The availability of herbicides in agricultural marketplaces plays a crucial role in determining farmers’ weed management practices and overall crop productivity. We surveyed the herbicide market by interviewing shopkeepers. The survey reveals various herbicide products in agrochemical shops across all 30 AEZs. With a total of 49 trade-named herbicides listed, farmers have access to a variety of options for weed control. These products encompass ten different herbicide formulations, with a notable presence of both single-active ingredients and combined-formulation herbicides. The availability of diverse formulations allows farmers to choose products tailored to their specific weed management needs and preferences.

Our data suggest regional variations in the popularity and availability of certain herbicides. Interestingly, this is not linked to farmers’ knowledge but to sellers’ promotions. Glyphosate and paraquat emerge as the most common herbicides used for pre-cultivation weed cleaning, with a significant presence in the market. However, there are notable differences in herbicide sales across AEZs, with higher quantities of glyphosate and paraquat sold in AEZ-29 than in other regions. Conversely, the absence of herbicide sales in AEZ-24 highlights their unsuitability among farmers. AEZs do not significantly influence the cost of herbicides; the price varies depending on the specific herbicide formulation and its brand. Triafamone, pyrazosulfuron ethyl, and bensulfuron methyl with acetochlor had higher prices, ranging from $0.80 to $2.08 per acre. These price differentials impact farmers’ purchasing decisions, making cost-effective options more appealing, especially for resource-constrained farmers. Our study highlights that herbicides are available throughout the country and season. Their accessibility to farmers depends on the farmer’s economic status and decision to apply herbicides. All herbicide products sold in the marketplaces are registered, indicating compliance with regulatory requirements. This ensures farmers can access approved and regulated herbicides undergoing safety and efficacy evaluations.

Our data highlight the cost-effectiveness of combining herbicides with manual weeding compared to relying solely on manual labor. This is primarily attributed to the herbicide efficiency and high labor cost associated with manual weeding and the narrow window of time for weed control, leading to labor shortages and increased wages. In addition, the affordability of herbicides makes them a cost-effective solution for initial weed suppression. Farmers’ responses suggested that manual weeding alone would be financially unsustainable in most regions due to high labor costs. This implies that some form of weed control, potentially including herbicides, is crucial for profitable rice cultivation.

Most farmers believe that herbicides have a positive role in increasing their rice harvest. They attribute this increase to their overall agricultural strategies, including herbicide application that enables timely and efficient weeding, significantly reducing competition between weeds and rice plants. On the other hand, farmers who disagree with herbicides increasing yield usually do not think of herbicides as having a positive impact on yield or growing plants. Rather than seeing herbicides as a tool to increase crop productivity, they might see them only as a way to control weeds. However, it is worth noting that some farmers perceived that manual weeding techniques could achieve comparable yields, albeit with labor availability and timely weeding challenges. The convenience and cost-saving benefits associated with herbicide application, particularly in reducing weeding costs, are widely recognized among respondents. Thus, the importance of timely weeding in getting high yields, for which herbicides have a positive and cost-effective role, is highlighted. However, nearly half of the farmers observed that herbicides negatively impact rice seedling growth. Herbicides inhibit the emergence of weed species from their propagules. However, they can also be toxic to rice plants, resulting in poor crop emergence, tillering, root damage, and potentially whiteheads. These issues typically arise when the herbicides are not used as recommended, such as when they are applied at the wrong rate or during the wrong stage of crop growth. The susceptibility of plants to damage varies depending on their variety, growth stage, and environmental conditions (Martini et al., 2022). Damage typically occurs shortly after the application of the herbicide. Toxic effects were noticed at an early stage of rice growth under treatment of various pre-emergence herbicides in a wide range of climatic conditions (Thapa et al., 2012; Tansay et al., 2021; Zhang et al., 2023). Farmers used additional fertilizers to alleviate such toxicity, imposing additional costs. Thus, research on mitigating toxicity would help develop effective plant protection strategies and maintain ecological balance (Barbaś et al., 2024). These include possibly integrating more post-emergence active substances (Juhász et al., 2024).

Only a small percentage of farmers follow product labels to obtain dosing and safety information. Although roughly 63% of farmers have completed at least elementary school, most of them are nevertheless unwilling to read herbicide labels that contain information about recommended dosages and possible side effects. Instead, most farmers rely on pesticide shops for guidance. However, this heavy reliance on shops can result in misinformation or overuse of herbicides, increasing costs and posing risks to health and the environment. A significant portion of the population learns from neighbors and friends, indicating the potential for community training.

Additionally, the low involvement of government extension workers suggests a lack of effective communication channels for disseminating accurate information and promoting best practices in herbicide use. Therefore, training programs for farmers are needed to create awareness. This strategy can reduce the possibility of overuse and its detrimental effects on the environment and crop health, ensuring the safe and efficient use of herbicides.

Most farmers do not report immediate health issues due to herbicide applications. However, a substantial number consider these to be potentially harmful. Despite recognizing the benefits of personal protective equipment (PPE), only a few farmers use it. Only a tiny proportion express concerns about soil health and environmental risks.

Identifying difficult-to-eradicate weeds from all AEZs would enhance our understanding of weed management issues, including herbicide dosage, resistance, and potential measures. During the survey, farmers also expressed their opinions on possible interventions. They reported the limited efficacy of pre-emergence herbicides on certain weeds, necessitating additional manual weeding that increases rice production costs. This highlights the need for more effective weed control strategies, including broader-spectrum herbicides. Considering the uncovered issues, farmers show a strong interest (99%) in genetically engineered rice varieties with tolerance to broad-spectrum herbicides, provided they are safe, profitable, and high yielding.

The survey also suggests that farmers are open to adopting the AWD irrigation method for water conservation. It has been reported that AWD could increase the farmers’ income by up to 32% in Bangladesh (Lampayan et al., 2015). However, weed concerns deter them from implementing AWD. Multiple studies reported that farmers perceived yield increases from using AWD (Kürschner et al., 2010; Rahman, 2015). The availability of an appropriate weeding method was recognized as the primary limiting factor in adopting AWD. The introduction of rice varieties tolerant to broad-spectrum post-emergence herbicides could incentivize AWD adoption, leading to water savings and cost reduction.

## 5 Conclusion

Currently, high labor prices make manual hand weeding very expensive in Bangladesh. Our comprehensive survey reflects the varied patterns of weed management practices, specifically herbicide application, and the prevalent challenges farmers face across 30 AEZs in Bangladesh. Our study concludes that herbicide-free rice cultivation is not feasible due to intense weed competition and high labor costs. Commonly used herbicides, such as pyrazosulfuron ethyl and a combination of bensulfuron methyl with acetochlor, effectively control weeds but can negatively impact rice seedling growth. This necessitates additional fertilization, which increases costs for farmers. Several weeds are not affected by pre-emergence herbicides. Our data highlight the potential benefits of introducing herbicide-tolerant rice cultivars. In addition, cultivating such crops will reduce water and tillage requirements, which can help conserve soil moisture and improve soil health. They would also allow flexibility in herbicide application. Overall, herbicide-tolerant rice varieties offer multiple benefits for farmers and food security.

## Data Availability

The original contributions presented in the study are included in the article/Supplementary Material; further inquiries can be directed to the corresponding author.
