# Supplementary material for: Beyond yield: Unveiling farmer perceptions and needs regarding weed management in Bangladesh
Source: Front Bioeng Biotechnol. 2024 Oct 11;12:1410128. doi: 10.3389/fbioe.2024.1410128 (PMC11513551; doi:10.3389/fbioe.2024.1410128)
Supplement: Supplementary file 1 [file Table1.docx]

**Supplementary Table 1**. Chi-square test to find the association with different indicator with herbicide adoption

| **Association bet^n^** | **Pearson Chi Square** | **Phi value** | **Cramer’s V** |
| --- | --- | --- | --- |
| Herbicide adoption and hand-weeding adoption | 5.199* | -0.078 |  |
| Herbicide use and years of rice cultivation experience | 5.507 |  | 0.080 |
| Herbicide adoption and education level | 49.047*** |  | 0.246 |
| Herbicide use and negative impact on rice | 60.596*** | 0.265 |  |
| Herbicide use and negative impact on health | 71.892*** | 0.288 |  |
| Herbicide use and impact on the Environment | 145.636*** | 0.410 |  |
| Herbicide use and yield increment | 197.592*** | 0.478 |  |

**p < 0.05*, ***p < 0.001*, ****p<0.0001*

**Supplementary Table 2.** Point-biserial correlation to find the relationship between herbicide adoption and total farming area

|  | | Herbicide adoption | Farming land (Acre) |
| --- | --- | --- | --- |
| Herbicide adoption |  | 1 |  |
| Farming land (Acre) |  | -0.022 | 1 |

**Supplementary Table 3.** Paired sample t-test to find the differences in terms of cost reduction for farmers while adopting herbicide

|  | t | Effect size |
| --- | --- | --- |
| The total cost of the combination of herbicide and hand weeding - Total cost of Hand weeding | 21.707*** | 0.81 |

****p<0.0001*

**Supplementary Table 4**: Multinomial logistic regression results for weed management strategy adopted by the rice farmers.

| Variable | Characteristics | Only hand-weeding vs. Only herbicide | | Herbicide-hand weeding combination vs. Only herbicide | |
| --- | --- | --- | --- | --- | --- |
|  |  | B | O.R. | B | O.R. |
| Total cost of herbicide |  | -0.007* | 0.993 | -0.007* | 0.993 |
| Total cost for weed management |  | 0.001 | 1.001 | 0.001 | 1.001 |
| Number of herbicide application |  | -14.952** | 3.209E-07 | -4.297* | 0.014 |
| Number of hand weeding |  | 7.006* | 1102.775 | 7.841* | 2542.574 |
| Is there any negative effect of herbicide on rice | Yes | - | - | - | - |
|  | No | -5.924* | 0.003 | -3.968 | 0.019 |
| Do herbicides increase yield | Yes | - | - | - | - |
|  | No | 2.886 | 17.929 | 2.038 | 7.678 |
| Any negative effect of herbicide on environment | Yes | - | - | - | - |
|  | No | 1.900 | 6.683 | .769 | 2.158 |

Model Fit: Chi-square (df 14) = 944.95*** Pseudo R-square 0.665^a^, 0.958^b^

Notes: Beta coefficients (B) and Odds Ratios (O.R.) shown. **p < 0.05*, ***p < 0.001*, ****p<0.0001* (2-tailed tests). ^a^Cox and Snell and ^b^Nagelkerke Pseudo R-squared reported.

**Supplementary Table 5:** Cost of weed control in an herbicide-manual weeding combination in comparison with full-manual weeding scenario. Average values are provided based on farmers’ response in 30 agro-ecological zones.

*Considering the scenario if no herbicides are used and weeding is performed only by manual hand weeding

| **AEZ** | **Current cost ($/acre)** | | **Total** | **Projected cost ($/acre)^*^** | **Cost savings due to herbicides (%)** |
| --- | --- | --- | --- | --- | --- |
|  | **Herbicide cost** | **Labor cost** |  |  |  |
| 1 | 2.41 | 23.51 | 25.92 | 70.54 | 63.25 |
| 2 | 2.89 | 54.29 | 57.18 | 162.89 | 64.89 |
| 3 | 2.93 | 26.29 | 29.22 | 78.87 | 62.95 |
| 4 | 7.10 | 38.46 | 45.56 | 115.37 | 60.51 |
| 5 | 4.64 | 41.01 | 45.65 | 119.01 | 61.64 |
| 6 | 3.16 | 8.88 | 12.04 | 26.48 | 54.53 |
| 7 | 2.99 | 76.98 | 79.97 | 140.21 | 42.96 |
| 8 | 3.09 | 36.15 | 39.24 | 108.45 | 63.82 |
| 9 | 2.79 | 35.28 | 38.07 | 105.83 | 64.03 |
| 10 | 1.68 | 13.68 | 15.36 | 36.04 | 57.38 |
| 11 | 1.95 | 31.54 | 33.49 | 94.64 | 64.61 |
| 12 | 1.32 | 76.66 | 77.98 | 87.45 | 10.83 |
| 13 | 4.09 | 85.53 | 89.62 | 181.62 | 50.66 |
| 14 | 4.08 | 50.30 | 54.38 | 157.71 | 65.52 |
| 15 | 3.88 | 29.62 | 33.50 | 70.31 | 52.35 |
| 16 | 8.64 | 19.28 | 27.92 | 36.14 | 22.74 |
| 17 | 1.82 | 113.55 | 115.37 | 307.095 | 62.43 |
| 18 | 2.55 | 60.04 | 62.59 | 143.78 | 56.47 |
| 19 | 4.76 | 41.31 | 46.07 | 123.93 | 62.83 |
| 20 | 2.79 | 42.34 | 45.13 | 85.98 | 47.51 |
| 21 | 2.01 | 24.07 | 26.08 | 33.68 | 22.57 |
| 22 | 2.09 | 23.93 | 26.02 | 39.09 | 33.44 |
| 23 | 1.82 | 45.49 | 47.31 | 62.49 | 24.29 |
| 24 | 0.0 | 23.13 | 23.13 | 23.13 | 0 |
| 25 | 1.98 | 30.67 | 32.65 | 92.02 | 64.52 |
| 26 | 1.89 | 30.95 | 32.84 | 92.85 | 64.63 |
| 27 | 4.01 | 22.99 | 27.00 | 67.96 | 60.27 |
| 28 | 1.52 | 38.69 | 40.21 | 90.10 | 55.37 |
| 29 | 1.58 | 49.99 | 51.57 | 143.49 | 64.06 |
| 30 | 4.79 | 27.17 | 31.96 | 60.08 | 46.80 |

**Supplementary Table 6.** Eigenvalues and the percentage of variance explained by each component

| **Component** | **Eigenvalues** | | |
| --- | --- | --- | --- |
|  | **Total** | **% of Variance** | **Cumulative %** |
| 1 | 2.466 | 16.441 | 16.441 |
| 2 | 1.807 | 12.044 | 28.484 |
| 3 | 1.565 | 10.432 | 38.916 |
| 4 | 1.248 | 8.318 | 47.235 |
| 5 | 1.182 | 7.883 | 55.118 |
| 6 | 0.985 | 6.566 | 61.684 |
| 7 | 0.908 | 6.055 | 67.739 |
| 8 | 0.873 | 5.818 | 73.558 |
| 9 | 0.792 | 5.277 | 78.835 |
| 10 | 0.754 | 5.028 | 83.862 |
| 11 | 0.676 | 4.510 | 88.372 |
| 12 | 0.588 | 3.920 | 92.292 |
| 13 | 0.517 | 3.446 | 95.738 |
| 14 | 0.381 | 2.540 | 98.278 |
| 15 | 0.258 | 1.722 | 100.000 |
