# Supplementary material for: Beyond yield: Unveiling farmer perceptions and needs regarding weed management in Bangladesh
Source: Front Bioeng Biotechnol. 2024 Oct 11;12:1410128. doi: 10.3389/fbioe.2024.1410128 (PMC11513551; doi:10.3389/fbioe.2024.1410128)
Supplement: Supplementary file 2 [file DataSheet1.docx]

Supplementary Figure 1: Common weeds resilient to pre-emergence herbicides in rice cultivation across various AEZs.
